# SdPI, The First Functionally Characterized Kunitz-Type Trypsin Inhibitor from Scorpion Venom

**DOI:** 10.1371/journal.pone.0027548

**Published:** 2011-11-08

**Authors:** Ruiming Zhao, Hui Dai, Su Qiu, Tian Li, Yawen He, Yibao Ma, Zongyun Chen, Yingliang Wu, Wenxin Li, Zhijian Cao

**Affiliations:** State Key Laboratory of Virology, College of Life Sciences, Wuhan University, Wuhan, People's Republic of China; MRC National Institute for Medical Research, United Kingdom of America

## Abstract

**Background:**

Kunitz-type venom peptides have been isolated from a wide variety of venomous animals. They usually have protease inhibitory activity or potassium channel blocking activity, which by virtue of the effects on predator animals are essential for the survival of venomous animals. However, no Kunitz-type peptides from scorpion venom have been functionally characterized.

**Principal Findings:**

A new Kunitz-type venom peptide gene precursor, SdPI, was cloned and characterized from a venom gland cDNA library of the scorpion *Lychas mucronatus*. It codes for a signal peptide of 21 residues and a mature peptide of 59 residues. The mature SdPI peptide possesses a unique cysteine framework reticulated by three disulfide bridges, different from all reported Kunitz-type proteins. The recombinant SdPI peptide was functionally expressed. It showed trypsin inhibitory activity with high potency (K_i_ = 1.6×10^−7^ M) and thermostability.

**Conclusions:**

The results illustrated that SdPI is a potent and stable serine protease inhibitor. Further mutagenesis and molecular dynamics simulation revealed that SdPI possesses a serine protease inhibitory active site similar to other Kunitz-type venom peptides. To our knowledge, SdPI is the first functionally characterized Kunitz-type trypsin inhibitor derived from scorpion venom, and it represents a new class of Kunitz-type venom peptides.

## Introduction

Venomous animals are considered to be a very distinctive class of species among animals. Evolution has equipped them with venom glands and venoms, that provide remarkable advantages for their survival [1], [2]. Various venomous animals such as snakes, spiders, sea anemones, cone snails, and scorpions are not only dangerous but also attractive to human beings. Their lethal venoms contain a diversity of bioactive proteins and peptides, which are important for predation and have proved to be of utility for informing drug design [1], [2].

Kunitz-type venom peptides constitute one such group of peptides and were named after the conserved Kunitz motif present in bovine pancreatic trypsin inhibitor (BPTI). The Kunitz-type polypeptide usually consists of 50 to 60 amino acid residues and has a disulfide rich alpha/beta fold structure. Most of them possess one conserved active site which plays an important rule in inhibiting the function of proteases. The structure/function relationships of Kunitz-type venom peptides have been extensively studied since they were first isolated from snake venoms [3], [4]. Kunitz-type venom peptides are usually comprised of 60 amino acids. They possess a relatively conserved active site loop region [5]. Currently reported Kunitz-type venom peptides can be classified into two families based on different cysteine frameworks. One family retains the typical Kuntiz-type architecture, with three highly conserved disulfide bridges, exemplified by HWTX-XI from spider, DTX-K from snake, and kalicludines from sea anemone [6], [7], [8]. The other family has only four cysteine residues, which results in the apparent ‘loss’ of a conserved disulfide bridge, represented by conkunitzin-S1 from cone snail [9]. Although distinct in primary structure, Kunitz-type venom peptides usually have potency as a protease inhibitor or potassium channel blocking function, or both. They were postulated to evolve from “old” body protein families and to play important roles both in protecting other venomous toxins from degradation or in blocking potassium channels in the prey [7]. Recently, more new functions of Kunitz-type venom peptides have been identified, such as voltage-gated sodium channels inhibition or analgesic activity [10], [11].

Until now, Kunitz-type venom peptides have been isolated from a wide variety of venomous animals [12]. However, few Kunitz-type venom peptides have been functionally characterized from scorpions, a widely distributed family of species. Prior to our work, only one putative Kunitz-type carboxypeptidase inhibitor from the Mexican scorpion *Hadrurus gertschi* was reported [13]. *Lychas mucronatus* is a scorpion species found in southern China. Recently, our group collected a population of *Lychas mucronatus* from Yunnan province and constructed its venom gland cDNA library [14]. As a result of the venom gland cDNA library transcriptome analysis, a number of seldom reported toxins were characterized, including two putative Kunitz-type venom peptides with an apparently unique cysteine framework [15]. They were designated scorpion-derived protease inhibitor (SdPI) and SdPI-2. We have successfully expressed, purified, and characterized the Kunitz-type venom peptide SdPI. Synthetic chromogenic substrate assay results demonstrated that SdPI is a potent inhibitor for trypsin and possesses good thermostability. The structure/function relationships for SdPI were further revealed by using site-directed mutagenesis combined with computer-based molecular dynamics simulation. SdPI is the first functionally characterized Kunitz-type venom peptide derived from scorpion venom and represents a new Kunitz-type venom peptide family with protease inhibitory activity.

## Results

### Cloning and sequence analysis of SdPI cDNA

**Figure 1:**
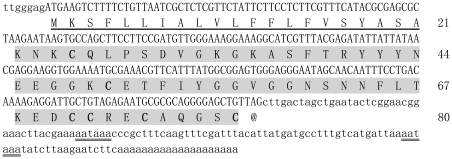
Precursor nucleotide sequence and deduced amino acid sequence of SdPI. The predicted protein sequence is shown below the nucleotide sequence. The 5′ and 3′ UTR regions are written in lower case letters. The signal peptide is underlined. The mature peptide is highlighted in gray color and the cysteine residues are shown in bold type. The potential polyadenylation signal is underlined twice.

Using random screening and bioinformatics analysis of the *Lychas mucronatus* venom gland cDNA library, a number of new scorpion venom toxins were identified [16]. After searching for homologues in the GenBank NCBI database, two putative Kunitz-type venom peptides differing in only two amino acids were found. One was termed SdPI (GenBank Accession No. GT028613). SdPI has a precursor nucleotide sequence of 364 nucleotides (nt) including three parts: 5′ untranslated region (UTR), open reading frame (ORF), and 3′UTR. The 5′UTR part is only 7 nt long. The ORF region of 243 nt encodes a precursor polypeptide of 80 amino acid residues including a 21-residue signal peptide and a 59-residue mature peptide. The 3′UTR is 114 nt long, and two aataaa polyadenylation signals were found 70 nt and 16 nt upstream of the poly(A) tail at the 3′UTR end (Figure 1).

### Primary structure analysis of SdPI

**Figure 2:**
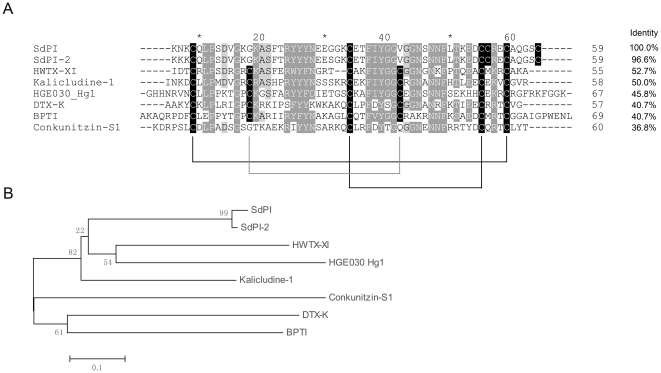
Sequence comparison and phylogenetical analysis of SdPI and other Kunitz-type proteins. (**A**) Sequence alignment of SdPI and other Kunitz-type proteins. Identical and similar residues are highlighted by black and gray colors. Predicted disulfide connections are shown with lines. The sequence identities of different Kunitz-type proteins compared with SdPI are shown on the right side. HWTX-XI is a bifunctional toxin isolated from spider. It can block voltage-sensitive K^+^ channels and inhibit trypsin activity. Kalicludine-1 is also a bifunctional toxin isolated from sea anemone. DTX-K is a snake derived Kunitz-type venom peptide with a potent potassium channel blocking function. Conkunitizin-S1 is a potassium channel inhibitor isolated from cone snail. HGE030_Hg1 from the Mexican scorpion *Hadrurus gertschi* is the only reported hypothetical scorpion-derived Kunitz-type venom peptide. BPTI is the first Kunitz-type protein, identified from bovine pancreas. (**B**) A minimum evolution (ME) tree of representative Kunitz-type proteins based on the multiple sequence alignment.

Multiple sequence alignments showed that mature SdPI shares homology with typical Kunitz-type venom peptides, including HWTX-XI from spider [7], kalicludine-1 from sea anemone [6], DTX-K from snake [8], and conkunitizin-S1 from cone snail [17]. The most homologous sequence HWTX-XI shows 52.7% identity to SdPI. However, compared to these typical Kunitz-type venom peptides, some obvious differences are observed (Figure 2A). Most of these Kunitz-type venom peptides possess a native Kunitz architecture involving three disulfide bonds, except conkunitizin-S1 which lacks the normal CysII–CysIV (CysII indicates the second cysteine in the primary structure of the peptide) disulfide on the surface of the molecule [9]. Interestingly, SdPI possesses a unique cysteine framework different from all reported Kunitz-type venom peptides. Like conkunitizin-S1, it also lacks the normal CysII–CysIV disulfide [18]. In addition, SdPI contains another two cysteine residues close to the C-terminus of the mature peptide. This special primary structure may generate a distinct disulfide connection (Figure 2B).

### Expression, purification, and characterization of recombinant SdPI (rSdPI)

**Figure 3:**
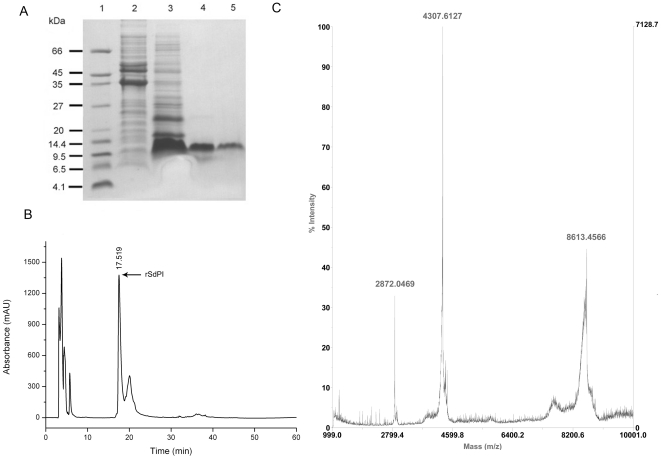
Purification and mass determination of rSdPI. (**A**) Tricine–SDS–PAGE analysis of expression and purification of rSdPI. Lane 1 shows molecular mass markers; Lanes 2 and 3 are the pellet fractions of mock-induced and IPTG-induced Rosetta (DE3) cells containing the expression plasmid pET-28a-SdPI, respectively; Lane 4 is refolded rSdPI after desalting and enrichment; Lane 5 is HPLC-purified rSdPI peptide. (**B**) Purification of rSdPI by RP-HPLC. The fractions containing rSdPI are indicated by arrows. (**C**) MALDI-TOF-MS mass spectrum of rSdPI. The predicted rSdPI mass is 8612.9 Da, and the measured value is 8612.5 Da.

The rSdPI peptide was produced as a fusion protein with a N-terminal His_6_-tag and a thrombin cleavage site [19]. After induction of the *E. coli* Rosetta (DE3) cell culture with isopropyl β-D-1-thiogalactopyranoside (IPTG), the rSdPI peptide was found exclusively in inclusion bodies. Using a refolding protocols described in the Methods section, soluble folded rSdPI was recovered (Figure 3A). After concentration, the soluble material was separated by reverse phase high-performance liquid chromatography (RP-HPLC). The peak eluting at 17.5 min corresponding to rSdPI peptide was collected (Figure 3B) and identified by matrix-assisted-laser-desorption/ionization time-of-flight mass spectrometry (MALDI-TOF-MS). Accounting for the loss of 6 Da from Cys thiol groups engaged in three disulfide bridges, the predicted molecular weight of the oxidized rSdPI peptide is 8612.9 Da. MALDI-TOF-MS showed a triply charged ion at m/z 2872.05, a doubly charged ion at m/z 4307.61, and a singly charged ion at m/z 8613.46, all corresponding to the same peptide with an average mass of 8612.5 Da, consistent with the calculated value (Figure 3C). The rSdPI peptide yield was 5.5 mg/L Luria Bertani (LB) media.

### Serine protease inhibitory activity of SdPI

**Figure 4:**
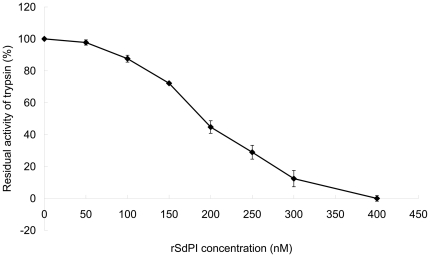
The trypsin inhibitory ability of different concentrations of rSdPI. The concentration dependence of trypsin inhibition is shown with different concentrations of rSdPI. Trypsin (final concentration 400 nM) was incubated with various concentration of rSdPI (0 to 400 nM) for 30 min. Data represent the mean ± S.E. of at least three experiments.

**Figure 5:**
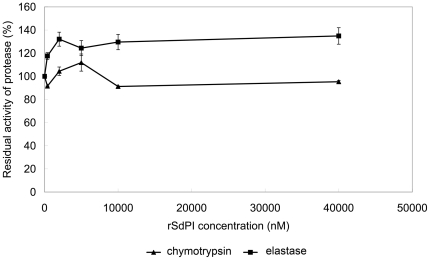
The inhibitory ability of different concentrations of rSdPI against chymotrypsin and elastase. The concentration dependence of chymotrypsin inhibition (closed triangles) and elastase inhibition (closed squares) is shown with different concentrations of rSdPI. Chymotrypsin or elastase (final concentration100 nM) were incubated with various concentration of rSdPI (0 to 40000 nM) for 30 min. Data represent the mean ± S.E. of at least three experiments.

**Figure 6:**
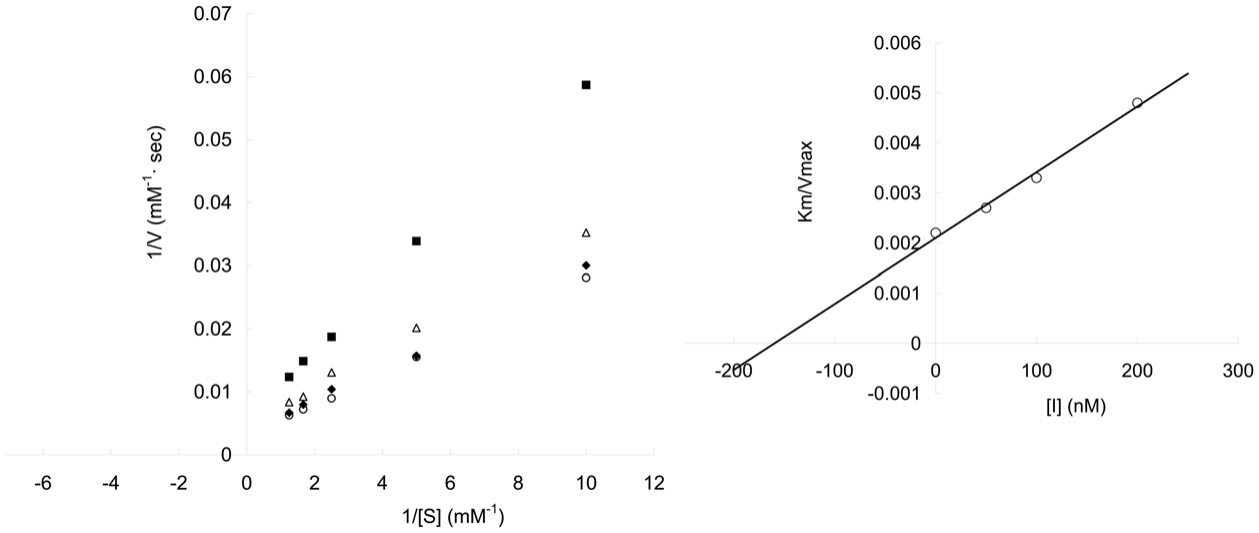
The inhibition of trypsin by rSdPI. Lineweaver-Burk plots for the determination of K_m_/V_max_ values of trypsin activity on a synthetic chromogenic substrate in the absence (○) or presence of 50 nM (⧫), 100 nM (△) and 200 nM (▪) rSdPI, respectively. (Inset) Secondary plot: the slopes (K_m_/V_max_) of the primary Lineweaver-Burk graphs were plotted against the concentration of inhibitor. The inhibitory constant (K_i_) is determined from the intercept point on the x-axis.

The purified rSdPI peptide was assayed for inhibitory activity against trypsin, chymotrypsin, and elastase by measuring the inhibition of hydrolysis of synthetic chromogenic substrates by serine proteases. The results showed that the rSdPI peptide inhibited trypsin with a 1∶1 stoichiometric ratio (Figure 4), but exhibited no inhibitory effect on chymotrypsin and elastase even at high concentration (Figure 5). Furthermore, the inhibitory constant (K_i_) of the trypsin/SdPI complex was determined by Lineweaver-Burk plots and further slope replotting, yielding a K_i_ value of 1.6×10^−7^ M (Figure 6).

### Identification of rSdPI functional residues

**Figure 7:**
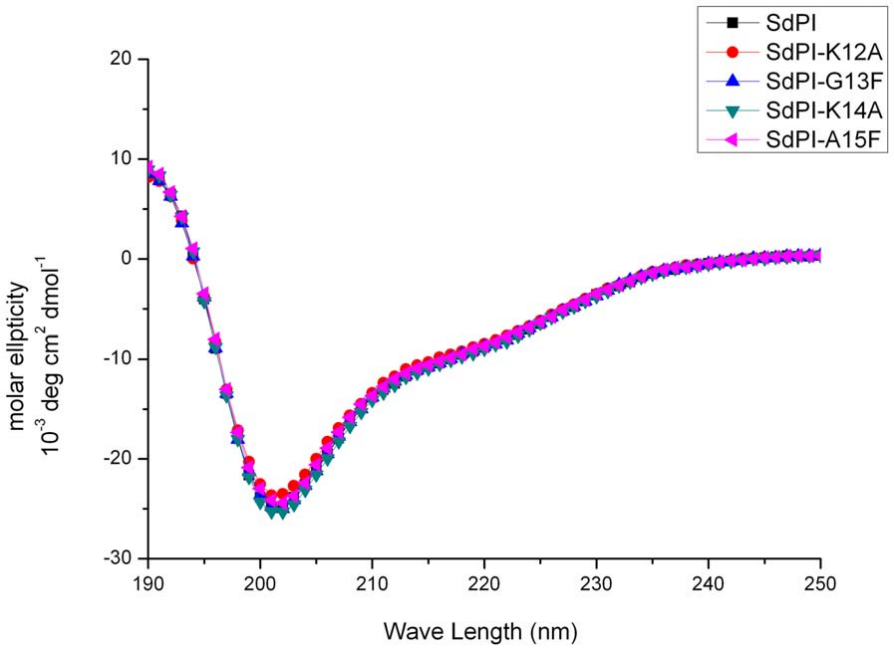
Circular dichroism spectrum analysis of rSdPI and site-directed mutants. The CD spectrum of SdPI and mutants was measured in the UV range 190–250 nm at a concentration of 0.2 mg/ml in water at 25°C.

**Figure 8:**
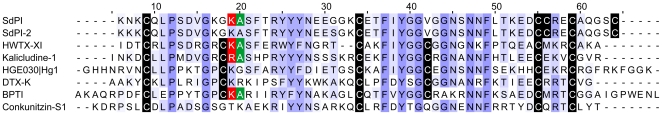
Active site comparison for SdPI and other Kunitz-type proteins. Identical and similar residues are highlighted in blue. Cysteine residues are highlighted in black. The predicted P1 positions of the active site of Kunitz-type proteins with protease inhibition activity are highlighted in red and the P1' positions in green.

Although the primary amino acid sequence of SdPI shows extensive homology with other Kunitz-type venom peptides (for example more than 50% identity with HWTX-XI and kalicludine-1), it adopts a distinct cysteine framework to stabilize its molecular structure. Most notably, in comparison to the typical Kunitz-type motif represented by BPTI, the conserved cysteine and proline residues in the P2 and P3 positions (the surrounding residues of the active site according to the distance from P1 position) are changed into glycine and lysine (Figure 2) [20]. To identify whether SdPI possesses a similar active site to other Kunitz-type venom peptides, four residues (Lys12, Gly13, Lys14, Ala15) close to the putative active site according to multiple sequence alignments and previous literature were targeted for mutagenesis [7], [21]. All mutants were expressed and purified by the same protocol described above for wild-type SdPI. Compared with that of the wild-type peptide, the circular dichroism (CD) spectrum of each of the mutants indicated no significant change in secondary structure, suggesting that they all adopted the same structural topology (Figure 7). The inhibitory constants (K_i_) of wild-type SdPI and four mutants against trypsin were measured and these values are listed in Table 1. The results showed that the Lys14Ala mutant displayed no inhibitory activity to trypsin up to 40 µM. Therefore, Lys14 likely corresponds to the P1 position (the key active site residue used to directly interact with the S1 pocket of protease). The adjacent residues apparently make minor contributions to the inhibitory activity since mutation has little effect on trypsin inhibition, though it is notable that the Ala15Phe mutation results in a nearly 400-fold decrease in inhibitory potency (Figure 8).

**Table 1:** Trypsin inhibitory activities of SdPI and its mutants.

Protein	WT	K12A	G13F	K14A	A15F
Inhibition constant, (K_i_) (M)	1.6×10^−7^	2.9×10^−7^	2.6×10^−7^	-	6.2×10^−5^

The inhibitory activities of SdPI and its mutants on the hydrolysis of synthetic chromogenic substrates by trypsin were assayed in 100 mM Tris-HCl (pH 8.0), containing 10 mM CaCl_2_ at 25°C. Trypsin was pre-incubated with the inhibitor for 30 min. The reaction was initiated by addition of synthetic chromogenic substrates. Formation of *p*-nitroaniline was monitored continuously at 405 nm for 5 min. Inhibition constants of SdPI and mutants were determined by Lineweaver-Burk plots and further replotting of the slopes. Errors in K_i_ values are less than ± 10%.

-, no inhibition detected.

**Figure 9:**
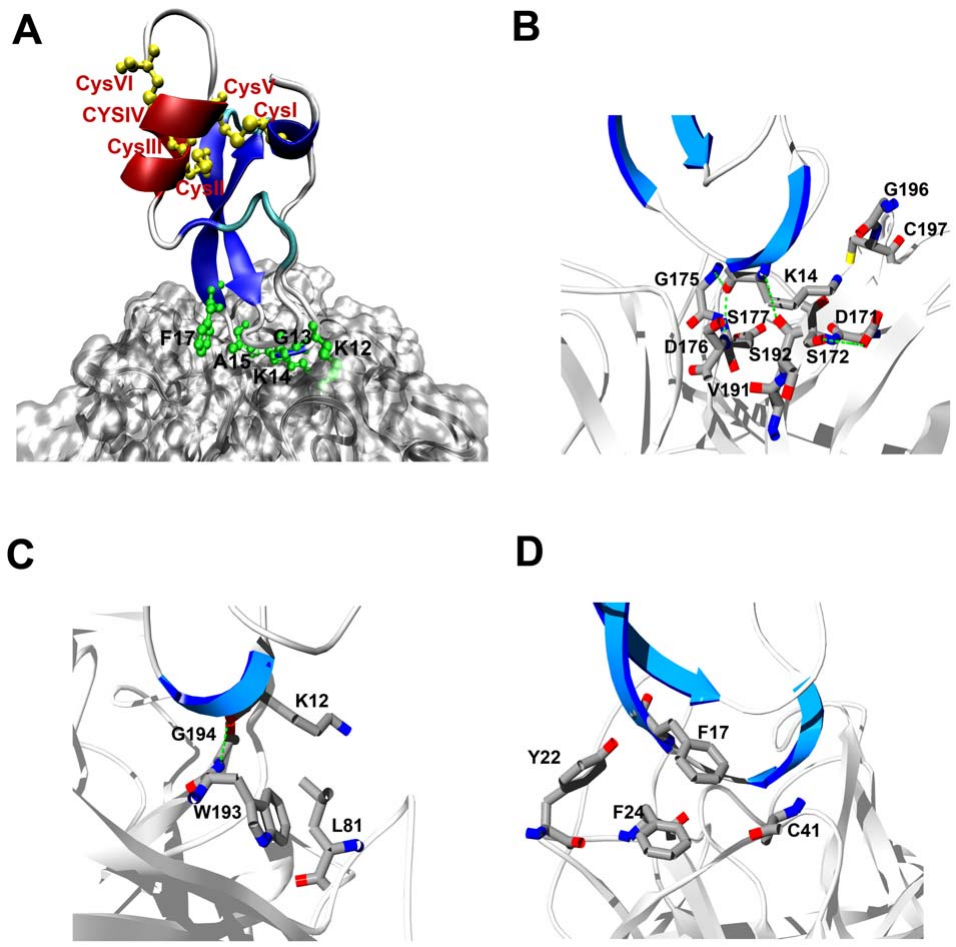
SdPI-trypsin complex predicted by molecular dynamics simulation. (**A**) The α-helix and β-strands of the modeled SdPI structure are displayed in red and blue, respectively. Disulfide bonds are shown in yellow. The active site residues of SdPI are represented as green sticks. (**B**) Lys14, the P1 residue of SdPI, can fit into the S1 pocket of trypsin. (**C**) The adjacent residue Lys12 likely also contributes to enhancing the SdPI-trypsin interactions. (**D**) The nearby residue Phe17 may also contribute to enhancing the SdPI-trypsin interaction.

To further examine the inhibition assay results, molecular dynamics (MD) simulation was employed to probe the stability of a proposed SdPI-trypsin complex model, in which the SdPI was set up to adopt a similar position to BPTI in the complex with trypsin (PDB accession code 2PTC). During the 2 ns simulation, the SdPI remained in a relatively stable position on the trypsin surface, with its putative active site formed by Lys12, Gly13, Lys14, and Ala15 inside the S1 pocket of trypsin (Figure 9A). In the coordinates at the end of the MD trajectory, strong polar and nonpolar interactions were observed among residues in the proximity of the active site. The SdPI Lys14 side chain protrudes inside the pocket formed by trypsin Asp171, Ser172, Gly175, Asp176, Ser177, Val191, Ser192, G196, and Cys197 (Figure 9B). Trypsin residues Asp176 and Ser192 were found to form hydrogen bonds with SdPI Lys14 (Figure 9B). Such strong polar interactions rationalize the complete loss of inhibitory activity after mutating Lys14 to alanine, supporting the prediction that Lys14 is located in the P1 position. The adjacent residues Lys12 and Phe17 also make their own contributions to enhancing the intermolecular interactions. Within a distance of 4 Å, SdPI Lys12 contacts trypsin Leu81, Trp193, and Gly194 (Figure 9C), whereas SdPI Phe17 mainly forms hydrophobic interactions with trypsin aromatic residues Tyr22 and Phe24 as well as Cys41 in this domain (Figure 9D). Therefore, although the SdPI apparently adopts a new Cys54-Cys59 disulfide bridge at the C-terminus (Figure 9A), it likely possesses a conserved overall structure, and a similar mode of trypsin interaction to other Kunitz-type venom peptides.

### Thermostability of SdPI

**Figure 10:**
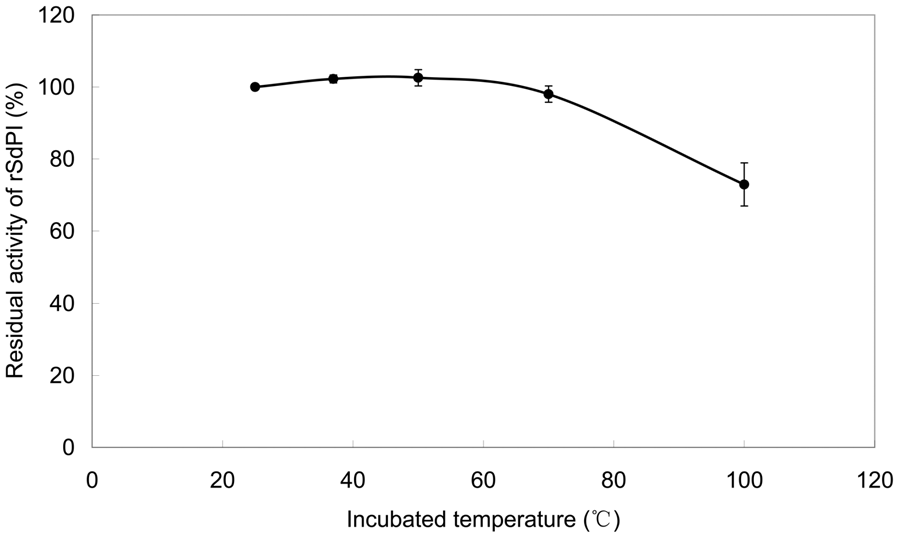
Inhibition of trypsin by SdPI at elevated temperatures. The residual trypsin inhibitory activity of rSdPI was determined after incubation for 1 hour at temperatures from 25 to 100°C. Data represent the mean ± S.E. of at least three experiments.

To test the thermostability, aliquots of the purified recombinant SdPI peptide were incubated for 1 hour in preheated test tubes at several temperatures up to 100°C, and the residual trypsin inhibitory activity was then determined (Figure 10). The result showed that there was about 73% residual trypsin inhibitory activity remaining after incubation at 100°C for 1 hour, indicating that SdPI is a thermostable protein.

## Discussion

To date, Kunitz-type venom peptides have been isolated from almost all the well-known venomous animals [7]. But hardly anything is known about this type of toxin in scorpion venom. In this work, a novel Kunitz-type venom peptide termed SdPI was identified in the scorpion *Lychas mucronatus*. We report here the cloning, expression, purification, and characterization of this novel scorpion-derived Kunitz-type venom peptide. Based upon a protease inhibition assay, SdPI was confirmed to be a potent trypsin inhibitor. This result supports the idea that Kunitz-type venom peptides are an essential toxin group, present in nearly all familiar venomous animals.

Moreover, SdPI possesses a unique cysteine framework different from any other Kunitz-type protein [17]. Compared to the typical Kunitz motif, SdPI lacks the normal CysII–CysIV disulfide but obtains another two cystine residues at the C-terminus. We used a computer-based molecular dynamics simulation to predict the SdPI 3D structure and the results showed that the change in cysteine positions may lead to the formation of a novel disulfide connection in the SdPI molecule, while allowing for a similar interaction model with trypsin compared to other Kunitz-type venom peptides. Site-directed mutagenesis results are also consistent with the computer prediction. These characteristics suggest that SdPI is representative of a new family of Kunitz-type venom peptides. Although the physiological target of Kunitz-type serine protease inhibitors in venom is still unclear, it is conceivable that these proteins play an important role in the survival of venomous animals, perhaps by protecting toxin peptides from degradation or generating a synergistic effect with other neurotoxins [7].

Presently, it is acknowledged that most toxin types were recruited into the venom proteomes from “old” protein families during the evolutionary process [22]. Subsequently, adaptive evolution following gene duplication made a primary contribution to the diversification of toxin types [23]. Frequently, new functions have been grafted onto old protein scaffolds [24]. Kunitz-type venom peptides represent one typical protein family that accords with the suggested recruitment pattern and its evolutionary origin has been well discussed in snake venom [25]. With respect to SdPI, Darwinian selection pressures may have forced the peptide to keep its activity against proteases but to also generate two other cysteine residues far away from the active site. These changes form a different disulfide connection in the molecule, potentiating a specific, as yet unknown function. All these characteristics suggest SdPI could be a useful molecule for elaborating the evolution and diversification of venom toxins.

Trypsin is reported to be involved in many inflammatory reactions in the human body, such as pancreatitis and other cardiovascular and nervous systems diseases [26], [27]. Trypsin inhibitors, such as ulinastatin and aprotinin, are already being clinically used in anti-inflammatory therapy [28]. Venomous animals are a rich source of protease inhibitors. Their divergent venom peptides are still waiting for exploitation in drug development. Scorpion-derived Kunitz-type venom peptide SdPI is a potent trypsin inhibitor with a K_i_ value of 1.6×10^−7^ M. Compared with other Kunitz-type venom peptides, SdPI has greater trypsin inhibitory activity than snake-derived bungaruskunin, but weaker activity than sea anemone-derived kalicludines and spider-derived HWTX-XI (Table 2) [6], [7], [29]. Although more study is needed to explain why these different Kunitz-type venom peptides vary in activity, they are nonetheless potential candidates for engineering more specific inhibitors against trypsin.

**Table 2:** Trypsin inhibitory activities of SdPI and other Kunitz-type venom peptides derived from venomous animals.

Source	Species	Name	K_i_ (M)
Snake	*B. fasciatus*	Bungaruskunin	9.8×10^−4^
Spider	*O. huwena*	HWTX-XI	2.3×10^−10^
Sea anemone	*A. sulcata*	kalicludines	3.0×10^−8^
Scorpion	*L. mucronatus*	SdPI	1.6×10^−7^

Neurotoxins from venom are valuable molecular probes for studying the role of ion channels in disease [30]. However, these neurotoxic peptides are easily degraded in many situations. Based on the thermostability assay, SdPI is a stable scorpion venom peptide. Previous research indicated that mutation of just a few residues may introduce new ion channel blocking functions into a Kunitz-type motif [10]. Considering that SdPI is a stable Kunitz-type venom peptide with selective trypsin inhibitory activity, its sequence could be an excellent template for future scientific study and molecular design.

Scorpion venoms are combinatorial peptide libraries of numerous toxin types with extreme diversity. Previous studies have identified a large number of toxins using bioassay-guided isolation methods and “-ome” approaches [31]. However, current research still mainly focuses on two classes of functional molecules: typical neurotoxins and antimicrobial peptides [32], [33]. Other atypical toxins in scorpion venom are seldom studied. Our work on SdPI indicates that even low abundance toxins, such as Kunitz-type venom peptides, could be both important for scorpion survival and useful for disease studies.

## Materials and Methods

### cDNA library construction and screening

The *Lychas mucronatus* were obtained from Jiufang small towns in Shidian county in Yunnan province of China and artificially fed in a simulated wild habitat in the laboratory. All animal studies were approved by the Institutional Animal Care and Use Committee at Wuhan University. Venom glands of 60 wild specimens were removed 2 days after extraction of their venom by electrical stimulation and ground into fine powder in liquid nitrogen [15]. The total RNA of the venom glands of *Lychas mucronatus* was isolated with TRIZOL Reagent (Invitrogen, U.S.A.), and then mRNA was prepared with the PolyATtract® mRNA Isolation Systems (Promega, U.S.A.). SuperScript™ Plasmid System (Invitrogen, U.S.A) was used to construct the cDNA library. cDNA inserts were directionally cloned into the plasmids pSPORT 1, following the manufacturer's instructions. After transforming the recombinant plasmids into electrocompetent *Escherichia coli*, random colonies were selected for nucleotide sequencing using an ABI 3730 automated sequencer.

### Bioinformatic analysis of the cDNA library and Kunitz-type toxins

Sequences were identified for open reading frames using ORFfinder (http://www.ncbi.nlm.nih.gov/projects/gorf/). After excluding signal peptides, the similarity was analysed by searching against GenBank NCBI database (http://www.ncbi.nlm.nih.gov/blast) using BLAST algorithms. All the sequence alignments were performed with Clustal_X 1.83 software followed by manual adjustment and viewed with the software Jalview. The multiple sequence alignment was used to carry out phylogenetic analysis using MEGA3.1.

### Construction of expression vector pET-28a-SdPI

The cDNA sequence of SdPI from *Lychas mucronatus* venom gland cDNA library was used as the template for constructing the protein expression vector. Primers were designed to match the mature peptide region of SdPI. The forward primer was 5′-GCGCAGCATATGAAGAATAAGTGCCAGCTTC-3′ (*Nde*I restriction site underlined). The reverse primer was 5′-CTGCGGATCCTCAACAGCTCCCCTGCGCGCAT-3′ (*Bam*HI restriction site underlined). The PCR product was digested with *Nde*I and *Bam*HI, and then inserted into the cut pET-28a vector. After verification by DNA sequencing, the recombinant plasmid pET-28a-SdPI was transformed into *E. coli* Rosetta (DE3) cells for expression.

### Site-directed mutagenesis

QuikChange® Site-Directed Mutagenesis Kit (Stratagene, U.S.A.) was used for generating the mutants based on the wild-type plasmid pET-28a-SdPI. All plasmids of mutants were verified by DNA sequencing before expression.

### Expression, purification and characterization of SdPI and its mutants

Cells transformed with expression plasmids of SdPI and mutants were cultured at 37°C in LB medium with 30 µg/ml kanamycin and 34 µg/ml chloramphenicol. Protein synthesis was induced by the addition of 0.75 mM IPTG when the optical density at 600 nm reached 0.3. After incubation for 4 hours at 37°C, 1 L of cell culture was centrifuged. Cell pellets were resuspended in phosphate-buffered saline (PBS) and lysed by sonication on ice. The recombinant SdPI protein was found to accumulate exclusively in inclusion bodies, and so was refolded *in vitro* using the following procedures. The insoluble inclusion bodies were first washed twice with 1% (v/v) Triton X-100 in PBS and then denatured in 5 ml 6 M guanidinium hydrochloride, 0.1 M Tris-HCl (pH 8.0), 1 mM EDTA, 30 mM reduced glutathione. The rSdPI was reactivated by 100-fold dilution in renaturation solution [0.2 M ammonium acetate (pH 7.5), 0.2 mM oxidized glutathione] at 16°C for 24 h. The soluble material was desalted and concentrated using centrifugal filter devices (cutoff value >5 kDa) (Sartorius Stedim Biotech, Germany). Renatured protein was finally purified by RP-HPLC on a C18 column (10×250 mm, 5 µm) (Elite-HPLC), using a linear gradient from 5% to 95% acetonitrile with 0.1% TFA in 60 min with a constant flow rate of 5 ml/min. Peaks of eluted protein were detected at 230 nm. The fraction containing rSdPI peptide eluted as major peaks at 30–33% acetonitrile, which were collected manually and immediately lyophilized. The molecular mass of the purified rSdPI peptide was further analyzed by MALDI-TOF-MS (Applied Biosystems).

The secondary structures of SdPI and its mutants were analyzed by CD spectropolarimetry. All purified peptides were dissolved in water at a concentration of 0.2 mg/ml. Spectra from 250 to 190 nm were recorded at 25°C with a scan rate of 50 nm/min on a Jasco-810 spectropolarimeter. The final CD spectra were obtained by averaging three scans and subtracting the signal from a water blank.

### Serine protease inhibition assays

The inhibitory activity of rSdPI and its mutants was tested by measuring the hydrolysis of synthetic chromogenic substrates in the presence serine proteases. Trypsin (bovine pancreatic trypsin; EC 3.4.21.4), chymotrypsin (bovine pancreatic α-chymotrypsin; EC 3.4.21.1), elastase (porcine pancreatic elastase; EC 3.4.21.36), and the chromogenic substrates N_α_-benzoyl-L-arginine 4-nitroanilide hydrochloride, N-succinyl-Ala-Ala-Pro-Phe p-nitroanilide, and N-succinyl-Ala-Ala-Ala-p-nitroanilide, were purchased from Sigma (U.S.A). The trypsin assay was performed in 100 mM Tris-HCl (pH 8.0) containing 10 mM CaCl_2_ in a total volume of 200 µl. Trypsin (final concentration was 400 nM) was incubated with various amounts of rSdPI or mutants (100 to 400 nM) for 30 min. The reactions were initiated by adding varying concentrations of substrate *N_α_*-benzoyl-L-arginine 4-nitroanilide hydrochloride ranging from 0.1 to 0.8 mM. The initial rate of *p*-nitroanilide (*p*NA) production was monitored continuously at 405 nm for 5 min at 25°C. The inhibitory activity of rSdPI was determined by setting the initial velocity with protease alone as 100% [29]. Lineweaver–Burk plots (1/V vs. 1/[S]) were used to determine the K_m_/V_max_ values of trypsin activity on *N_α_*-benzoyl-L-arginine 4-nitroanilide in the presence of different inhibitor concentration. The slopes (K_m_/V_max_) of curves were plotted against the concentration of inhibitor. The inhibitory constant (K_i_) of the trypsin/inhibitor complex can be determined from the intercept point of the secondary plot on the x-axis [19]. Inhibitory tests for chymotrypsin and elastase were carried out in the same manner as for trypsin, except with a lower protease final concentration of 100 nM and switching to the relevant chromogenic substrates.

### Atomic coordinates and molecular dynamics simulation

Molecular dynamics simulation was used for predicting the putative active site of SdPI. The atomic structure of SdPI was modeled by using the bovine pancreatic trypsin inhibitor (BPTI, PDB code: 1OA5) as a template. The structure of trypsin was extracted from the BPTI-trypsin complex (PDB code: 2PTC). Then a SdPI-trypsin complex was obtained through distance-restraint homologous modeling method [34] on the basis of the BPTI-trypsin complex and subjected to molecular dynamics simulation in explicit solvent to test its stability. To simulate SdPI with trypsin in explicit solvent, the starting complex was embedded in a periodic box containing 10250 TIP3P explicit water molecules, with a distance of 8.5 Å. The system was then subject to 400 ps equilibration and 2 ns unrestrained simulation using sander and PMEMD modules in the Amber8 program, respectively. The equilibration steps were taken by gradually reducing the force constant from 5.0 (kcal/mol)/Å^2^ for restraining all the heavy atoms to 0.02 (kcal/mol)/Å^2^ for backbone heavy atoms only. The temperature was set at 300K with a cutoff distance of 12 Å. The ff99 force field (Parm99) was applied in all the energy minimization and simulation steps.

### Thermostability assays

Aliquots of purified rSdPI peptide at a final concentration of 400 nM were dissolved in 100 mM Tris-HCl (pH 8.0) containing 10 mM CaCl_2_. Then the samples were added to preheated test tubes and incubated for 1 hour at temperatures from 25°C to 100°C [35]. The residual trypsin inhibitory activity of rSdPI peptide was determined by the method described above.
